# Exogenous H_2_S suppresses traumatic stress-induced changes of HMGB1 and ERS in rat liver and lungs

**DOI:** 10.1515/med-2026-1501

**Published:** 2026-09-29

**Authors:** Qingjie Zhang, Jianfeng Lu, Zhenyong Gu

**Affiliations:** Department of Police, Guangdong Justice Police Vocational College, Guangzhou, Guangdong, China; Department of Radiology, Guangzhou Medical University Affiliated Stomatological Hospital, Guangzhou, Guangdong, China; Department of Forensic Medicine, Nantong University, Nantong, Jiangsu, China

**Keywords:** hydrogen sulfide, HMGB1, endoplasmic reticulum stress, liver, lung, rat

## Abstract

**Objectives:**

Traumatic stress triggers systemic inflammation and organ injury via HMGB1 and endoplasmic reticulum stress (ERS), but whether H_2_S modulates this pathway remains unclear. This study aimed to investigate the effects of exogenous H_2_S (NaHS, an H_2_S donor) on traumatic stress-induced HMGB1 and ERS changes in rat liver and lungs.

**Methods:**

Male SD rats underwent bilateral hindlimb crush. Sixty rats were assigned to sham, crush, NaHS pretreatment (14, 28, 56 μmol/kg), delayed NaHS (120 μmol/kg), and NaHS-only groups (n=6). Serum ALT/AST, histopathology, HMGB1 (ELISA/IHC), and ERS markers (GRP78, caspase-12, CHOP, IRE1α) were assessed.

**Results:**

Crush increased ALT/AST, liver/lung injury, plasma HMGB1, cytoplasmic HMGB1 translocation, and ERS activation. NaHS at 28 μmol/kg offered strongest protection, reducing all parameters. The 14 μmol/kg dose had limited effect; 56 μmol/kg showed reduced efficacy and mild toxicity. Delayed 120 μmol/kg also attenuated injury and HMGB1–ERS activation, though less effectively than 28 μmol/kg pretreatment.

**Conclusions:**

Exogenous H_2_S attenuates traumatic stress-induced acute liver/lung injury, likely by inhibiting the HMGB1-mediated ERS pathway. Optimal protection occurs at 28 μmol/kg, while higher doses exhibit biphasic effects. These findings may inform forensic and critical care strategies.

## Introduction

Extensive soft tissue injury can induce remote organ damage through systemic inflammation, oxidative stress, and stress response pathways, leading to acute liver and lung injury, acute respiratory distress syndrome, or even traumatic multiple organ dysfunction syndrome (T-MODS). Acute liver and lung injury often occur early in T-MODS, and the interplay between these organs is complex, making the mechanisms of organ injury after trauma a critical topic in critical care and forensic medicine.

Hydrogen sulfide (H_2_S), the third endogenous gaseous signaling molecule, exerts vasodilatory, anti-inflammatory, antioxidative, and cytoprotective effects [1]. Previous studies have shown that H_2_S mitigates hepatic ischemia-reperfusion injury by reducing oxidative stress and apoptosis [2], and our laboratory has demonstrated that decreased endogenous H_2_S contributes to traumatic stress-induced acute liver injury in rats, whereas exogenous H_2_S alleviates this injury [3]. H_2_S also dynamically regulates cellular stress responses in a dose-dependent manner, suggesting both protective and potentially harmful effects depending on concentration [4].

Endoplasmic reticulum stress (ERS) plays a key role in liver and lung pathology by promoting cell death when protein homeostasis is disrupted [5]. High mobility group box-1(HMGB1), a redox-sensitive nuclear protein and late pro-inflammatory mediator, is released into circulation following trauma, amplifying inflammatory responses and contributing to remote organ injury [6]. Previous work indicates that HMGB1 upregulation acts upstream of ERS in traumatic stress-induced liver and lung injury [7], 8], while H_2_S may modulate HMGB1 activity through sulfhydration, altering its conformation, receptor interactions, and downstream signaling.

Based on these findings, we hypothesize that exogenous H_2_S can attenuate acute liver and lung injury induced by traumatic stress via the HMGB1–ERS pathway. To test this hypothesis, the present study addressed the following research questions: (1) Does exogenous H_2_S (NaHS) reduce HMGB1 expression/translocation and ERS activation in the liver and lungs of rats subjected to bilateral hindlimb crush? (2) What is the dose-response profile of NaHS (14, 28, 56 μmol/kg pretreatment and 120 μmol/kg delayed administration) in this model? (3) Is the protective effect of H2S associated with suppression of the HMGB1–ERS axis? The answers to these questions will help clarify the mechanism of H_2_S-mediated organ protection following traumatic stress and may provide a potential therapeutic target for forensic and critical care medicine.

## Materials and methods

### Experimental animals and reagents

A total of 60 male adult Sprague–Dawley (SD) rats (weighing 200 ± 25 g, specific pathogen-free grade) were obtained from Nantong University (Nantong, China). Animals were housed under controlled temperature (22 ± 1 °C) and a 12 h light/dark cycle, with food and water available *ad libitum*.

Sodium hydrosulfide (NaHS) was purchased from Sigma-Aldrich LLC (USA). Rabbit polyclonal antibodies against β-actin, GRP78, caspase-12, CHOP, and IRE1α were obtained from Santa Cruz Biotechnology (USA). Rabbit anti-rat polyclonal antibody against HMGB1 was purchased from Abcam (USA, clone not applicable, polyclonal). The rat HMGB1 ELISA kit (catalog No. PH406), bicinchoninic acid (BCA) protein assay kit, cell lysis buffer, hematoxylin and eosin staining solution, paraformaldehyde, chloral hydrate, and poly-L-lysine-coated slides were obtained from Beyotime Company (Shanghai, China).

### Preparation of models

#### Randomization and sample size

Sixty male SD were randomly assigned to 10 groups using a computer-generated random number sequence, with six rats per group (n=6). The sample size was determined based on preliminary experiments in our laboratory (unpublished data) and power analysis (α=0.05, β=0.2, effect size estimated from pilot studies).

#### Group allocation

The 10 groups were: (1) Sham, (2) crushing, (3–5) H_2_S pretreatment (14, 28, 56 μmol/kg NaHS, i.p., once daily for 3 days before crushing, plus one additional dose immediately after the 3-h compression), (6) H_2_S delayed administration (120 μmol/kg NaHS, i.p., immediately after decompression following the 3-h compression), and (7–10) H_2_S-only control groups (14, 28, 56, 120 μmol/kg NaHS, same injection schedule without crushing). Doses were chosen based on previous studies and our preliminary experiments [9], 10].These doses were chosen to span a range from sub-protective to mildly toxic, as H_2_S donors are known to exhibit a narrow therapeutic window with biphasic effects. The 28 μmol/kg dose has been reported to protect against trauma-induced organ injury in rats, while 56 μmol/kg approaches the threshold for mild cytotoxicity, and the higher 120 μmol/kg was selected for delayed intervention to overcome established post-traumatic inflammation [11], [12], [13].

#### Anesthesia and analgesia

Rats were anesthetized with intraperitoneal injection of 10 % chloral hydrate (0.4 ml/100 g). Chloral hydrate was used because of its rapid onset, short duration, and established reliability in this non-survival trauma model, as permitted by our institutional guidelines for acute terminal procedures. No additional analgesia was administered during the compression phase because the model is designed to simulate traumatic stress under anesthesia; however, all animals were monitored for signs of distress (respiratory rate, withdrawal reflexes) throughout the procedure. In accordance with our institutional animal care policy, any animal exhibiting severe distress would have been euthanized immediately, but no such event occurred.

#### Traumatic stress model

The rat model of traumatic stress was established by crushing both hindlimbs using a custom-made 15-kg standard weight. After satisfactory anesthesia, rats were placed prone on a grooved wooden board, and both hindlimbs (including the area below the tail root) were compressed continuously for 3 h, followed by three cycles of 30-min compression and 30-min decompression. The end of the final decompression was designated as time zero. Rats were returned to cages with clean food and were euthanized 18 h after the end of compression. Sham-operated rats underwent the same anesthesia and handling procedures without compression.

#### Blinding

Outcome assessments (histopathology, Western blot quantification, immunohistochemistry scoring, and biochemical assays) were performed by investigators blinded to group allocation. Tissue samples were coded, and group assignment was revealed only after data analysis was completed.

### Blood collection and biochemical assays

At the scheduled euthanasia time (18 h after compression), rats were deeply anesthetized, and blood (3–5 mL) was collected from the abdominal aorta using vacuum tubes with or without anticoagulant. Serum and plasma were separated by centrifugation (3,000 rpm, 10 min, 4 °C). Serum alanine aminotransferase (ALT) and aspartate aminotransferase (AST) activities were measured using an automatic biochemical analyzer (Hitachi 7600, Hitachi High-Tech, Tokyo, Japan; results expressed in U/L). Each sample was measured in duplicate, and the mean value was used for analysis. Plasma HMGB1 levels were determined using a rat HMGB1 ELISA kit (catalog No. PH406, Beyotime, Shanghai, China) according to the manufacturer’s instructions, with each sample run in duplicate.

### Tissue collection and processing

After blood collection, the liver and lungs were rapidly excised. The left lobe of the liver and the right lung were fixed in 4 % paraformaldehyde for histology; the right liver lobe and left lung were snap-frozen in liquid nitrogen and stored at −80 °C for protein analysis.

### Histopathology and scoring

Liver and lung tissues fixed in 4 % paraformaldehyde for 24 h were processed through routine dehydration, clearing, and paraffin embedding. Sections of 4 μm thickness were cut, mounted on poly-L-lysine-coated slides, and stained with hematoxylin and eosin (H&E) following standard protocols. Histopathological changes were observed under a light microscope (Olympus CX31, Tokyo, Japan) at ×200 magnification.

For liver injury, a semi-quantitative scoring system was used based on three parameters: congestion (0–4: 0=none, 1=mild, 2=moderate, 3=severe, 4=very severe), vacuolization (0–4 same scale), and necrosis (0–4). The total liver injury score was the sum of the three parameters (maximum 12), as described previously [14].

For lung injury, the following parameters were assessed on a 0–4 scale (0=absent, 1=mild, 2=moderate, 3=severe, 4=very severe): alveolar wall thickening, inflammatory cell infiltration, intra-alveolar hemorrhage, and edema. The total lung injury score was the sum of the four parameters (maximum 16). All scoring was performed by two independent pathologists blinded to group allocation, and the average score was used for statistical analysis [15].

### TUNEL staining (apoptosis detection)

Apoptotic cells in liver sections were detected using the terminal deoxynucleotidyl transferase dUTP nick end labeling (TUNEL) assay (One Step TUNEL Apoptosis Assay Kit, catalog No. C1088, Beyotime). Paraffin sections were dewaxed, rehydrated, and treated with proteinase K (20 μg/mL) for 20 min at 37 °C. After washing, sections were incubated with TUNEL reaction mixture for 60 min at 37 °C in the dark. Nuclei were counterstained with DAPI. TUNEL-positive cells (green fluorescence) were counted in five random high-power fields (×200) per section, and the average number per field was calculated.

### Immunohistochemistry (IHC) for HMGB1

Liver tissue sections (4 μm) were dewaxed, rehydrated, and subjected to antigen retrieval by heating in citrate buffer (10 mM, pH 6.0) in a microwave oven (95 °C, 15 min). Endogenous peroxidase activity was blocked with 3 % H_2_O_2_ for 10 min at room temperature. Sections were then incubated with rabbit anti-rat HMGB1 polyclonal antibody (Abcam, catalog No. ab18256; dilution 1:50) at 37 °C for 1 h, followed by overnight incubation at 4 °C. After washing, sections were incubated with MaxVision™ HRP-polymer reagent (anti-rabbit, Dako) for 30 min at room temperature. Diaminobenzidine (DAB) was used as the chromogen, and sections were counterstained with hematoxylin. Negative controls were performed by omitting the primary antibody or using rabbit IgG isotype control (Abcam, catalog No. ab172730) at the same concentration. Images were captured using a light microscope (Olympus CX31) at × 200 magnification. HMGB1 immunoreactivity was assessed based on staining intensity and subcellular localization (nuclear, cytoplasmic, or both).

### Western blotting

Frozen liver and lung tissues were homogenized in RIPA lysis buffer (Beyotime, catalog No. P0013B) containing protease and phosphatase inhibitors. Protein concentration was determined using the BCA assay (Beyotime). Equal amounts of protein (30 μg per lane) were separated by 10 % SDS-polyacrylamide gel electrophoresis (SDS-PAGE) and transferred onto polyvinylidene difluoride (PVDF) membranes (0.45 μm pore size, Millipore). Membranes were blocked with 5 % non-fat milk in Tris-buffered saline with 0.1 % Tween 20 (TBST) for 1 h at room temperature, then incubated overnight at 4 °C with primary antibodies: rabbit anti-HMGB1 (1:1,000, Abcam, ab18256), anti-GRP78 (1:1,000, Santa Cruz, sc-13968), anti-caspase-12 (1:1,000, Santa Cruz, sc-5626), anti-CHOP (1:1,000, Santa Cruz, sc-7351), anti-IRE1α (1:1,000, Santa Cruz, sc-20790), and anti-β-actin (1:2,000, Santa Cruz, sc-47778). After washing with TBST, membranes were incubated with horseradish peroxidase (HRP)-conjugated goat anti-rabbit secondary antibody (1:5,000, Beyotime, catalog No. A0208) for 2 h at room temperature. Protein bands were visualized using enhanced chemiluminescence (ECL) substrate (Millipore) and imaged with a Bio-Rad ChemiDoc MP system. Band intensities were quantified using Image Lab software (Bio-Rad), and target protein expression was normalized to β-actin.

### Statistical analysis

Data are presented as mean ± standard deviation (SD). Statistical analyses were performed using SPSS 26.0 (IBM, Armonk, NY, USA). Normality was tested using Shapiro–Wilk test, and homogeneity of variances was assessed by Levene’s test. One-way analysis of variance (ANOVA) followed by Bonferroni’s post hoc test was used for multi-group comparisons. Two-group comparisons were performed using two-tailed Student’s *t*-test. A p value<0.05 was considered statistically significant.

### Ethics statement

All animal experiments were approved by the Institutional Animal Care and Use Committee of Nantong University (Nantong, China) (Approval No. 2016-AE-056) and were conducted in accordance with the National Institutes of Health Guide for the Care and Use of Laboratory Animals (8th edition, 2011). This study adhered to the ARRIVE 2.0 guidelines for reporting animal research.

## Results

### Effects of H_2_S on trauma – induced liver injury, ERS, and HMGB1 expression in rats

#### H_2_S alleviates trauma – induced acute liver injury

Effects of H_2_S on serum ALT and AST activities in rats with trauma – induced stress: as shown in Figure 1, compared with the Sham group, the serum ALT and AST activities in the crushing group group were significantly increased (p<0.05), serum ALT increased from 44.7 ± 6.1 U/L (Sham) to 382.9 ± 58.5 U/L (crushing, p<0.05), and AST from 98.5 ± 10.2 U/L to 1,093.6 ± 85.4 U/L (p<0.05), by 701 and 1,011 % respectively. 14 μmol/kg NaHS had no significant effect on serum ALT and AST activities. 28 μmol/kg NaHS had no significant effect on serum ALT activity but could significantly reduce AST activity by 23 % (p<0.05). 56 μmol/kg NaHS significantly increased ALT and AST activities (p<0.05) by 45 and 54.5 % respectively. Compared with the crushing group, the serum ALT and AST activities in the 14 and 28 μmol/kg NaHS + crushing groups were significantly decreased (p<0.05), by 24 , 55, 60 % respectively. However, in the 56 μmol/kg NaHS + crushing group, there was no significant change in serum ALT, while AST activity was significantly decreased by 29 % (p<0.05). Compared with the Sham group, the serum ALT and AST activities in the 120 μmol/kg NaHS group were increased by 211 and 81 % respectively (p<0.05). Compared with the crushing group, after post – treatment with 120 μmol/kg NaHS, the serum ALT and AST activities in rats were significantly decreased by 57 and 60 % respectively (p<0.05).

**Figure 1:**
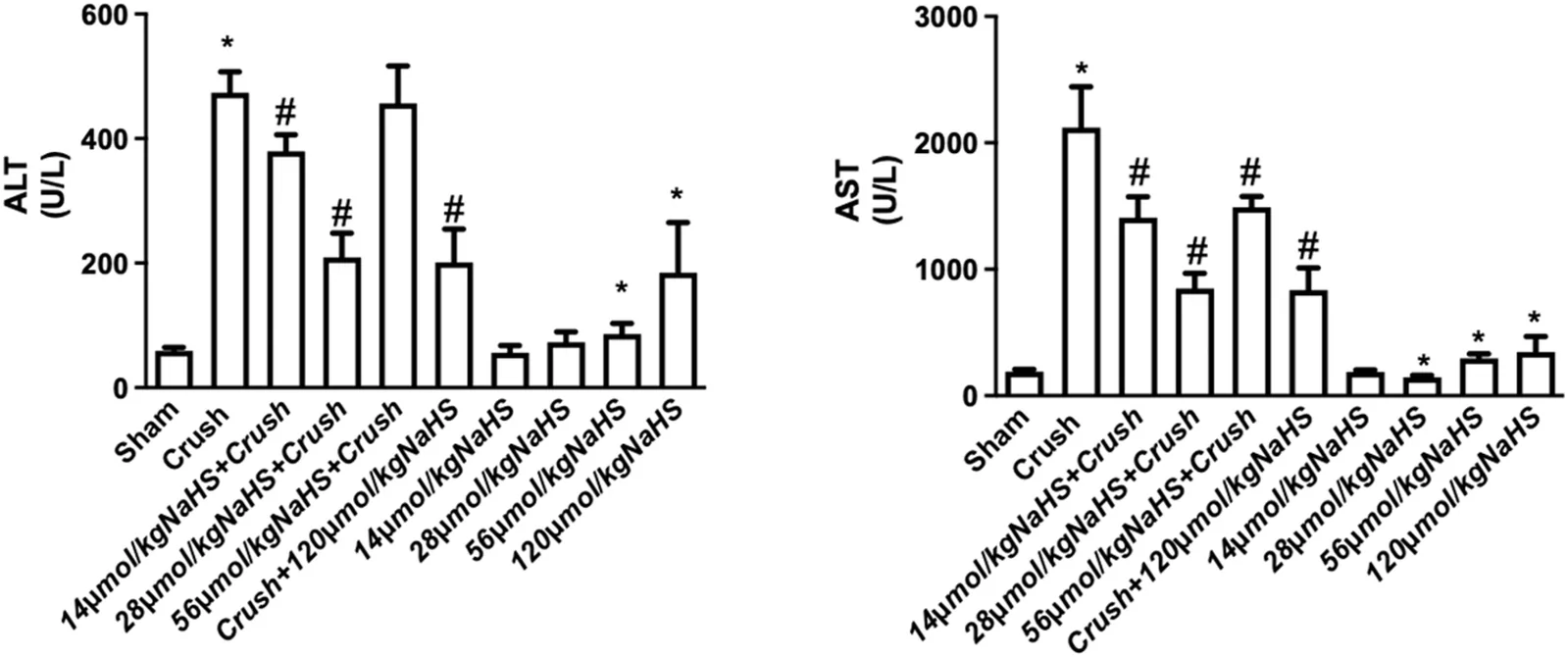
Effects of H_2_S dministration on changes in serum ALT and AST activities induced by traumatic stress in rats. *p<0.05 vs. Sham group; #p<0.05 vs. crushing group.

Effects of H_2_S on trauma – induced pathological changes in rat liver tissues: Figure 2 shows that the liver tissue structure in the Sham group was normal. In the crushing group, there was obvious congestion in the central vein and hepatic sinusoids, hydropic degeneration of hepatocytes, cytoplasmic vacuolation, and inflammatory cell infiltration in the hepatic sinusoids. Compared with the crushing group, the morphological changes such as congestion in the central vein and hepatic sinusoids of the hepatic lobule, swelling of hepatocyte volume, and loosening of cytoplasm in the 14, 28, and 56 μmol/kg NaHS + crushing groups and the crushing + 120 μmol/kg NaHS group were alleviated to varying degrees, among which the 28 μmol/kg NaHS pretreatment group and the 120 μmol/kg NaHS post – treatment group showed the most obvious alleviation. Compared with the Sham group, hepatocytes in the 56 μmol/kg NaHS group and the 120 μmol/kg NaHS group showed mild degeneration.

**Figure 2:**
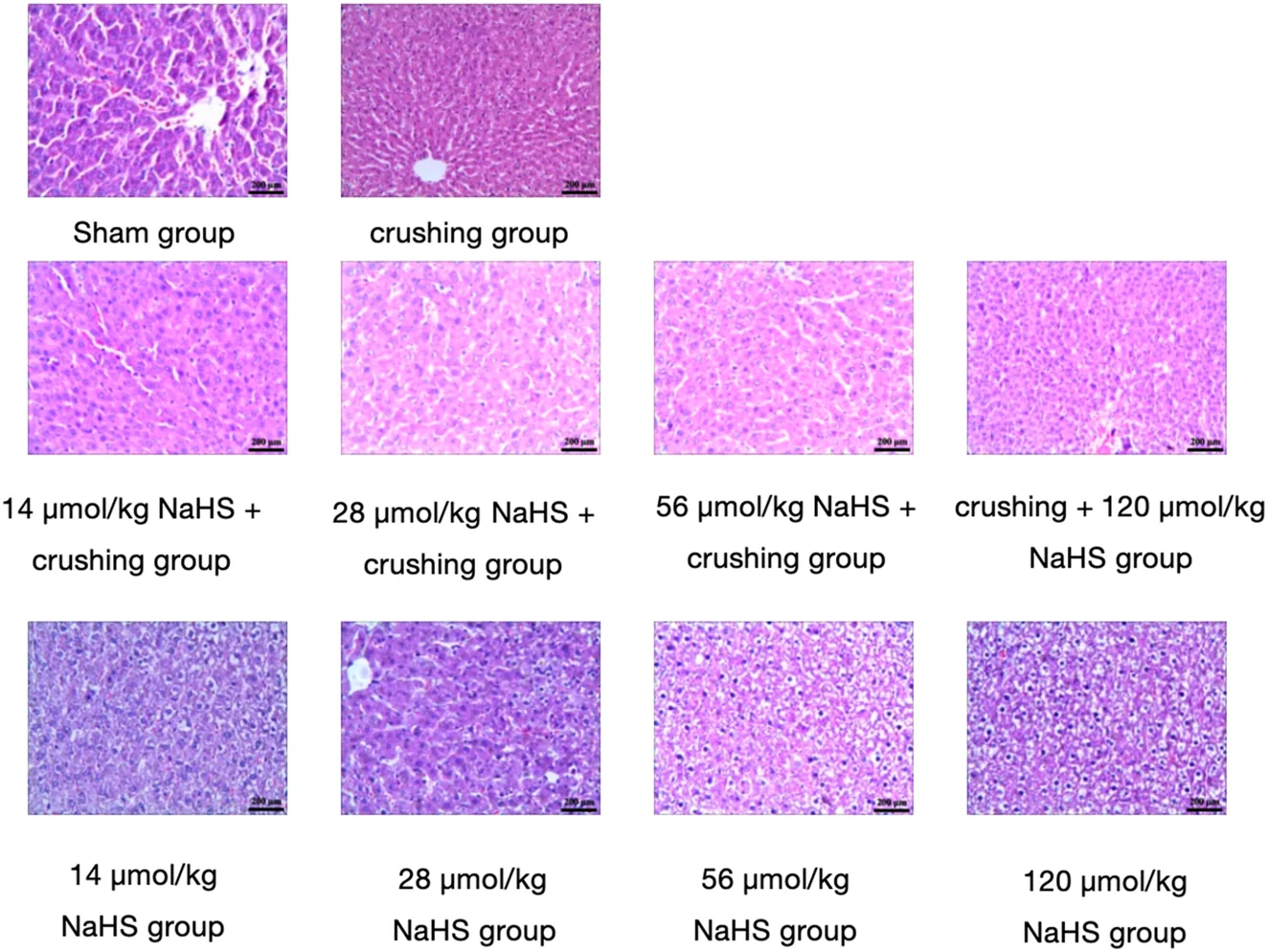
Pathological changes of rat liver tissues induced by trauma and affected by H_2_S assessed by HE staining (images were obtained at ×200 magnification, scale bar=200 µm).

Effects of H_2_S on trauma – induced increase in the number of TUNEL – positive cells in rat liver tissues:as shown in Figure 3, no TUNEL – positive cells were found in the liver tissues of rats in the Sham group. The number of TUNEL – positive cells in the liver tissues of rats in the crushing group increased significantly. Compared with the crushing group, the number of TUNEL – positive cells in the 14, 28, and 56 μmol/kg NaHS + crushing groups and the crushing + 120 μmol/kg NaHS group decreased to varying degrees, among which the 28 μmol/kg NaHS pretreatment group and the 120 μmol/kg NaHS post – treatment group showed the most obvious decrease. Compared with the Sham group, the number of TUNEL – positive cells in the liver tissues of rats in the 120 μmol/kg NaHS group increased significantly.

**Figure 3:**
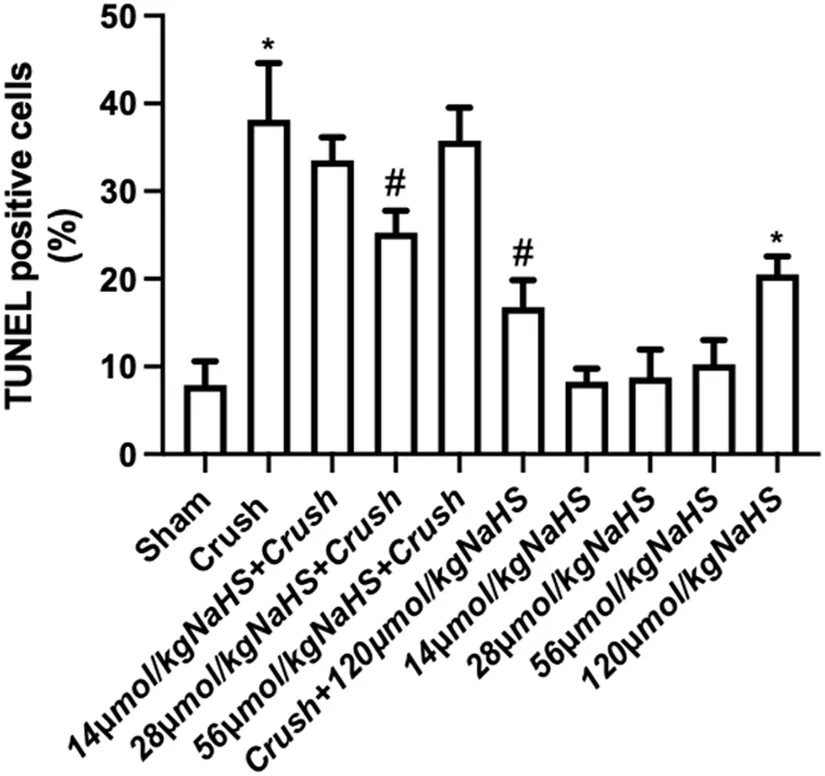
Effects of H_2_S on the increase in the number of TUNEL-positive cells in rat liver tissues induced by traumatic stress. *p<0.05 vs. Sham group; #p<0.05 vs. crushing group.

#### H_2_S alleviates endoplasmic reticulum stress (ERS) in rat liver tissues induced by traumatic stress

Effects of H_2_S pretreatment on ERS in rat liver tissues induced by traumatic stress: as shown in Figure 4, in the Sham group, there was a certain level of basal expression of ERS-related proteins GRP78, caspase-12, CHOP, and IRE1α in rat liver tissues. Compared with the Sham group, the expression of the aforementioned ERS-related proteins in the crushing group was significantly upregulated (p<0.05), increasing by 58 , 90, 190, and 114 % respectively. In the 14 and 28 μmol/kg NaHS groups, there were no significant changes in the expression of ERS-related proteins. In the 56 μmol/kg NaHS group, the expression of caspase-12 protein was significantly upregulated, increasing by 81 % (p<0.05). Compared with the crushing group, in the rat liver tissues pretreated with 14 μmol/kg NaHS, the expression of GRP78 and IRE1α proteins was significantly downregulated, decreasing by 20 and 45 % respectively (p<0.05), and the expression of caspase-12 and CHOP proteins showed a downward trend. In the rat liver tissues pretreated with 28 μmol/kg NaHS, the expression of the aforementioned ERS-related proteins was significantly downregulated (p<0.05), decreasing by 39 , 60, 52, and 68 % respectively. In the 56 μmol/kg NaHS pretreatment group, GRP78 still showed high expression, caspase-12 and CHOP showed a downward trend, while the expression of IRE1 protein was significantly reduced by 30 % (p<0.05).

**Figure 4:**
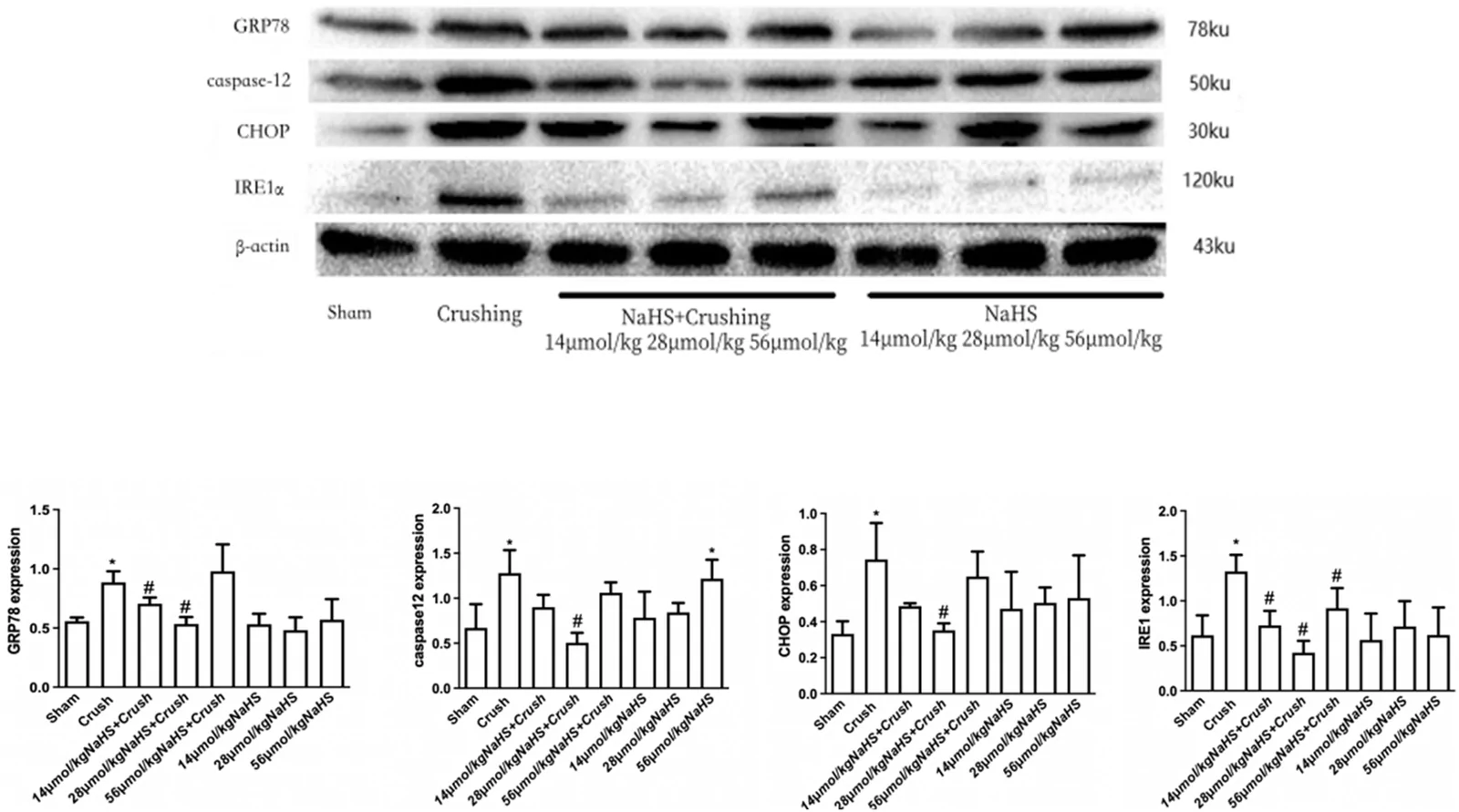
Effects of H_2_S pretreatment on ERS in rat liver tissues induced by traumatic stress. *p<0.05 vs. Sham group; ^#^p<0.05 vs. crushing group.

Effects of post-administration of H_2_S on ERS in rat liver tissues induced by traumatic stress: Figure 5 shows that compared with the Sham group, the expression of the aforementioned ERS-related proteins in the crushing group was significantly upregulated (p<0.05). In the 120 μmol/kg NaHS group, only the expression of IRE1α among the ERS-related proteins changed, increasing by 55 % (p<0.05), while there were no significant changes in GRP78, caspase-12, and CHOP.Compared with the crushing group, in the crushing + 120 μmol/kg NaHS group, the expression of the aforementioned ERS-related proteins in rat liver tissues was significantly downregulated, decreasing by 53 , 45, 52, and 42 % respectively (p<0.05).

**Figure 5:**
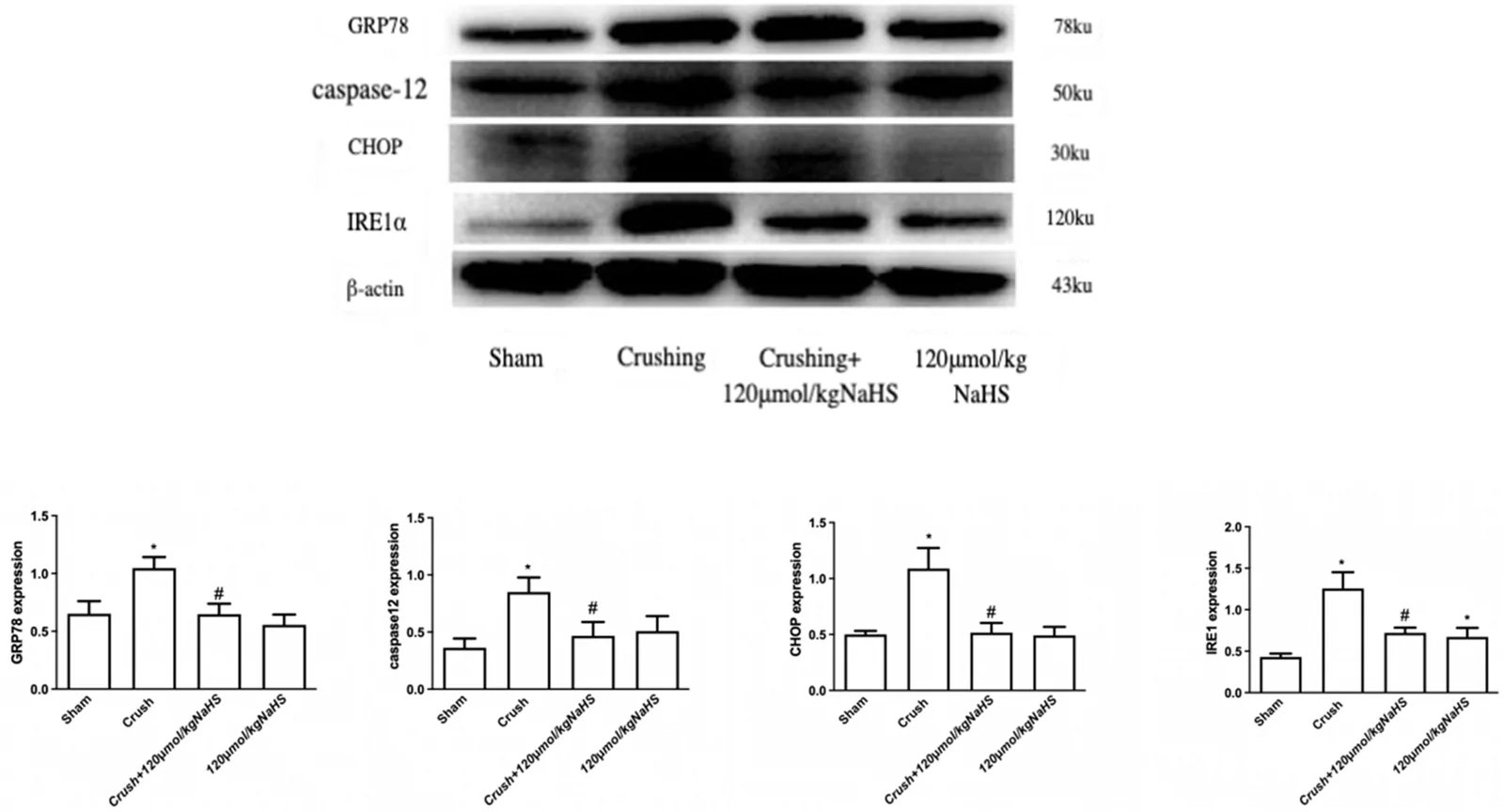
Effects of post-administration of H_2_S on ERS in rat liver tissues induced by traumatic stress. *p<0.05 vs. Sham group; #p<0.05 vs. crushing group.

#### H_2_S inhibits the increase in HMGB1 expression induced by traumatic stress in rat liver tissues

Effects of H_2_S on plasma HMGB1 levels in rats subjected to traumatic stress: as shown in Figure 6, a baseline level of HMGB1 was detected in the plasma of Sham group rats. Compared with the Sham group, the plasma HMGB1 content in the crushing group was significantly upregulated by 1,224 %. In the 14, 28, 56 and 120 μmol/kg NaHS groups, HMGB1 levels were also significantly increased (p<0.05) by 357 , 132, 175, and 410 % respectively.Compared with the crushing group, the plasma HMGB1 content in the 14, 28, and 56 μmol/kg NaHS + crushing groups was significantly reduced (p<0.05) by 68 , 86, and 81 %, respectively. Compared with the crushing group, the HMGB1 content in the 120 μmol/kg NaHS + crushing group was significantly downregulated by 85 %. The most pronounced reduction was observed in the 28 μmol/kg NaHS + crushing group.

**Figure 6:**
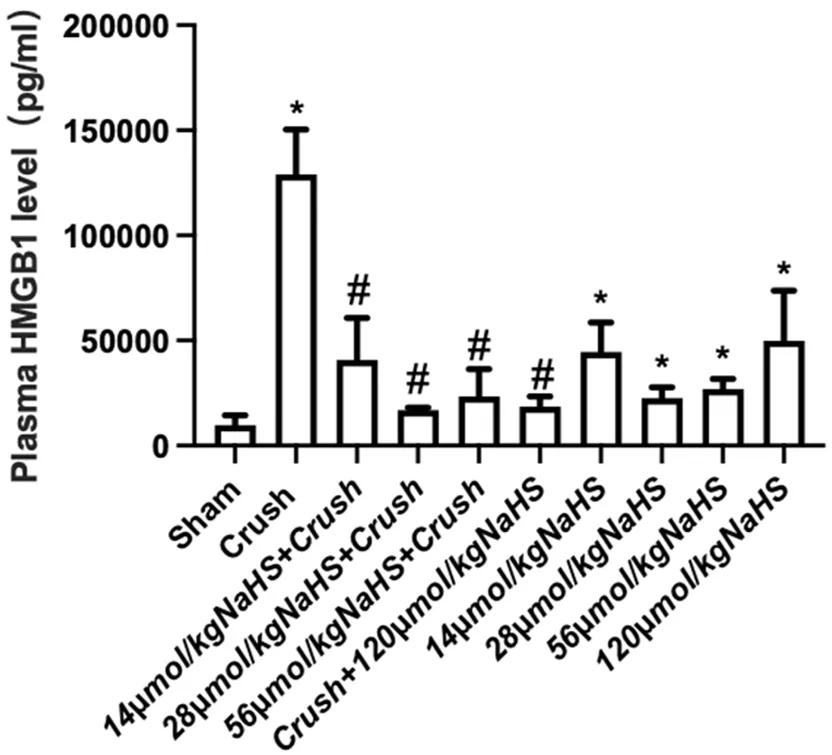
Effects of H_2_S on plasma HMGB1 level in rats with traumatic stress. *p<0.05 vs. Sham group; #p<0.05 vs. crushing group.

Effects of H_2_S pretreatment on HMGB1 expression in rat liver tissues induced by traumatic stress: baseline HMGB1 protein expression was detected in the liver tissues of Sham group rats. As shown in Figure 7, compared with the Sham group, HMGB1 expression in the crushing group was significantly upregulated by 104 %. No significant changes were observed in the 14 and 28 μmol/kg NaHS groups, while the 56 μmol/kg NaHS group showed a 75 % increase (p<0.05).Compared with the crushing group, HMGB1 expression in the 14 μmol/kg NaHS + crushing group remained unchanged, whereas the 28 μmol/kg NaHS + crushing group showed a significant 43 % reduction (p<0.05). The 56 μmol/kg NaHS + crushing group exhibited an upward trend but without statistical significance (p>0.05).

**Figure 7:**
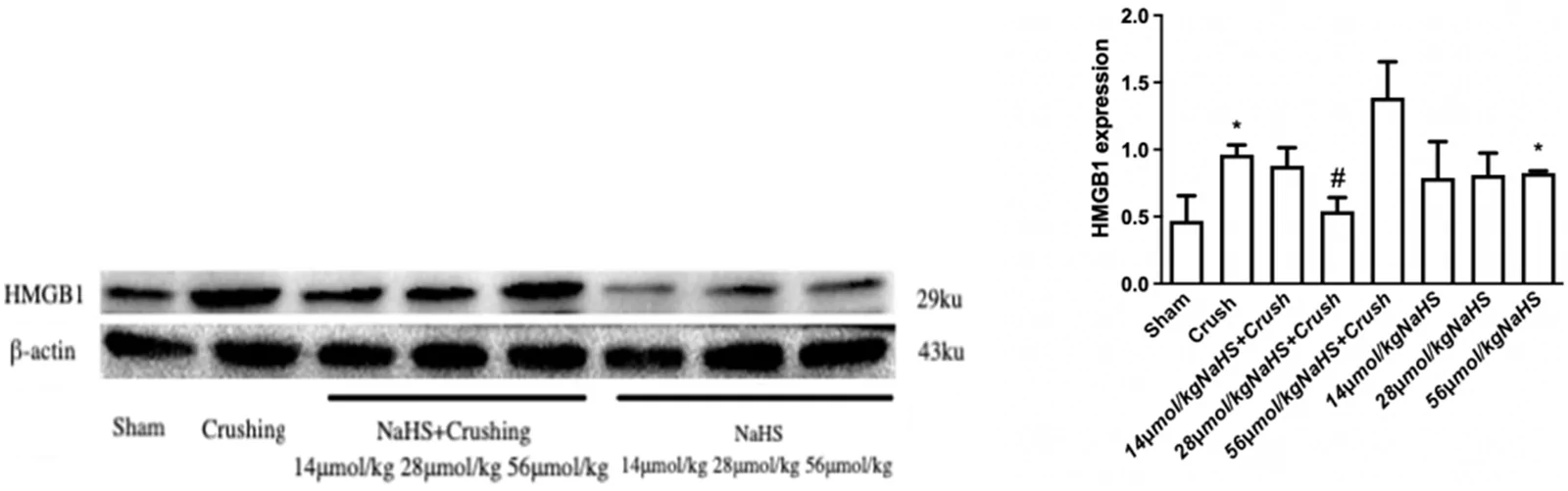
Effects of H_2_S pretreatment on HMGB1 expression in rat liver tissues with traumatic stress. *p<0.05 vs. Sham group; #p<0.05 vs. crushing group.

Effects of post-administration of H_2_S on HMGB1 expression in rat liver tissues induced by traumatic stress: as shown in Figure 8, compared with the Sham group, HMGB1 expression in the crushing group was significantly increased by 125 %, and the 120 μmol/kg NaHS group alone showed a 57 % increase (p<0.05).Compared with the crushing group, HMGB1 expression in the crushing + 120 μmol/kg NaHS group was significantly downregulated by 54 %.

**Figure 8:**
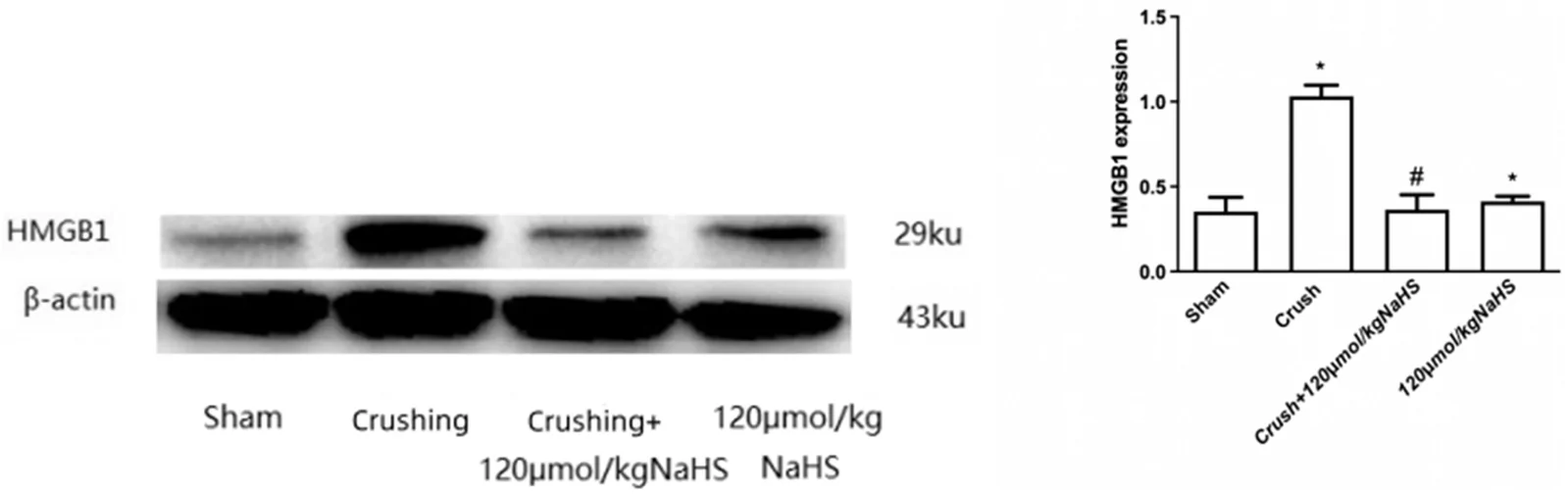
Effects of post-administration of H_2_S on HMGB1 expression in rat liver tissues with traumatic stress. *p<0.05 vs. Sham group; #p<0.05 vs. crushing group.

Effects of H_2_S on HMGB1 expression and cellular translocation in rat liver tissues induced by traumatic stress: Figure 9 shows that in the Sham group, HMGB1 was primarily localized in the nucleus of hepatocytes. In the crushing group, HMGB1 expression was significantly increased, with notable translocation from the nucleus to the cytoplasm.Compared with the crushing group, cytoplasmic HMGB1 expression was reduced in the 14, 28, and 56 μmol/kg NaHS + crushing groups and the crushing + 120 μmol/kg NaHS group, with the most significant effects observed in the 28 μmol/kg NaHS + crushing and crushing + 120 μmol/kg NaHS groups.

**Figure 9:**
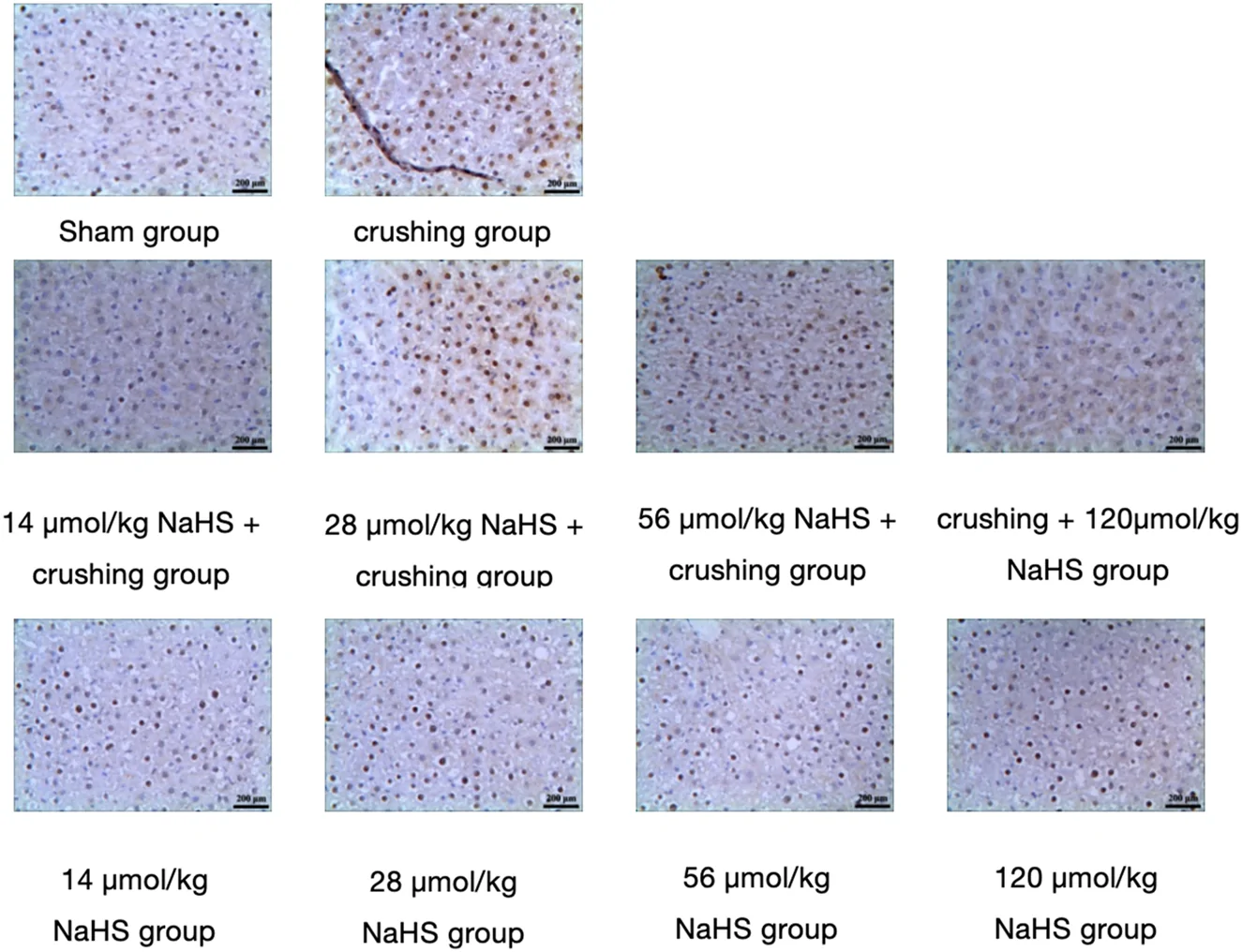
Effects of H_2_S on HMGB1 expression and cellular translocation in rat liver tissues with traumatic stress. Immunohistochemistry (images were obtained at ×200 magnification, scale bar=200 µm).

### Effects of H_2_S on trauma-induced lung injury, ERS, and HMGB1 expression in rats

#### H_2_S can alleviate trauma-induced acute lung injury in rats

Effects of H_2_S pretreatment on trauma-induced pathological changes in rat lung tissues:as shown in Figure 10, the lung tissue structure of rats in the Sham group was normal; the lung tissue lesions in the crushing group were obvious, with thickened alveolar walls and increased number of inflammatory cells; compared with the crushing group, the lung tissue lesions in the 14, 28, and 56 μmol/kg NaHS + crushing groups were significantly alleviated. The small blood vessels in the lung tissues of rats in the 14, 28, 56, and 120 μmol/kg NaHS groups showed mild dilation and congestion, presenting mild injury lesions.

**Figure 10:**
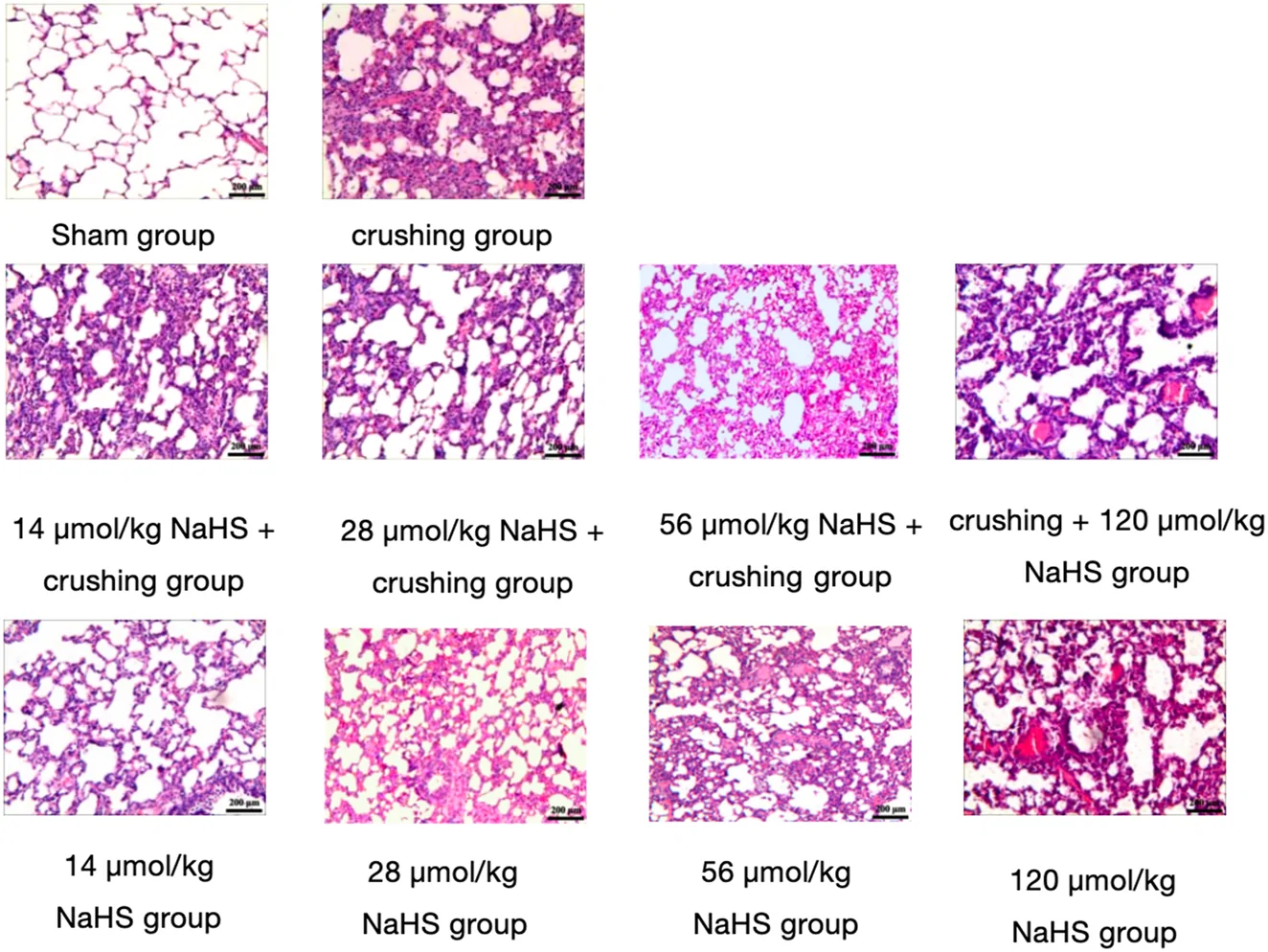
Pathological changes of rat lung tissues induced by trauma and affected by H_2_S assessed by HE staining (images were obtained at ×200 magnification, scale bar=200 µm).

#### H_2_S can alleviate trauma-induced ERS in rat lung tissues

Effects of H_2_S pretreatment on trauma-induced ERS in rat lung tissues:as shown in Figure 11, there was a certain amount of basal expression of ERS-related proteins GRP78, caspase-12, CHOP, and IRE1α in the lung tissues of rats in the Sham group; compared with the Sham group, the aforementioned ERS-related proteins in the lung tissues of rats in the crushing group were significantly upregulated (p<0.05), with increases of 34 , 50, 153, and 14 % respectively; compared with the Sham group, the GRP78 and CHOP proteins in the lung tissues of rats in the 14 and 28 μmol/kg NaHS groups were significantly upregulated, with increases of 52 , 88, 109 % respectively, and only the GRP78 protein in the lung tissues of rats in the 56 μmol/kg NaHS group was significantly upregulated, with an increase of 67 %; compared with the crushing group, the expression of CHOP protein in the lung tissues of rats in the 14 μmol/kg NaHS + crushing group was significantly downregulated, with a decrease of 28 %, and the expression of the aforementioned ERS-related proteins in the lung tissues of rats in the 28 μmol/kg NaHS + crushing group was significantly downregulated (p<0.05), with decreases of 24.3 , 39, 42, and 16 % respectively, while there was no significant change in the expression of the aforementioned ERS-related proteins in the 56 μmol/kg NaHS + crushing group.

**Figure 11:**
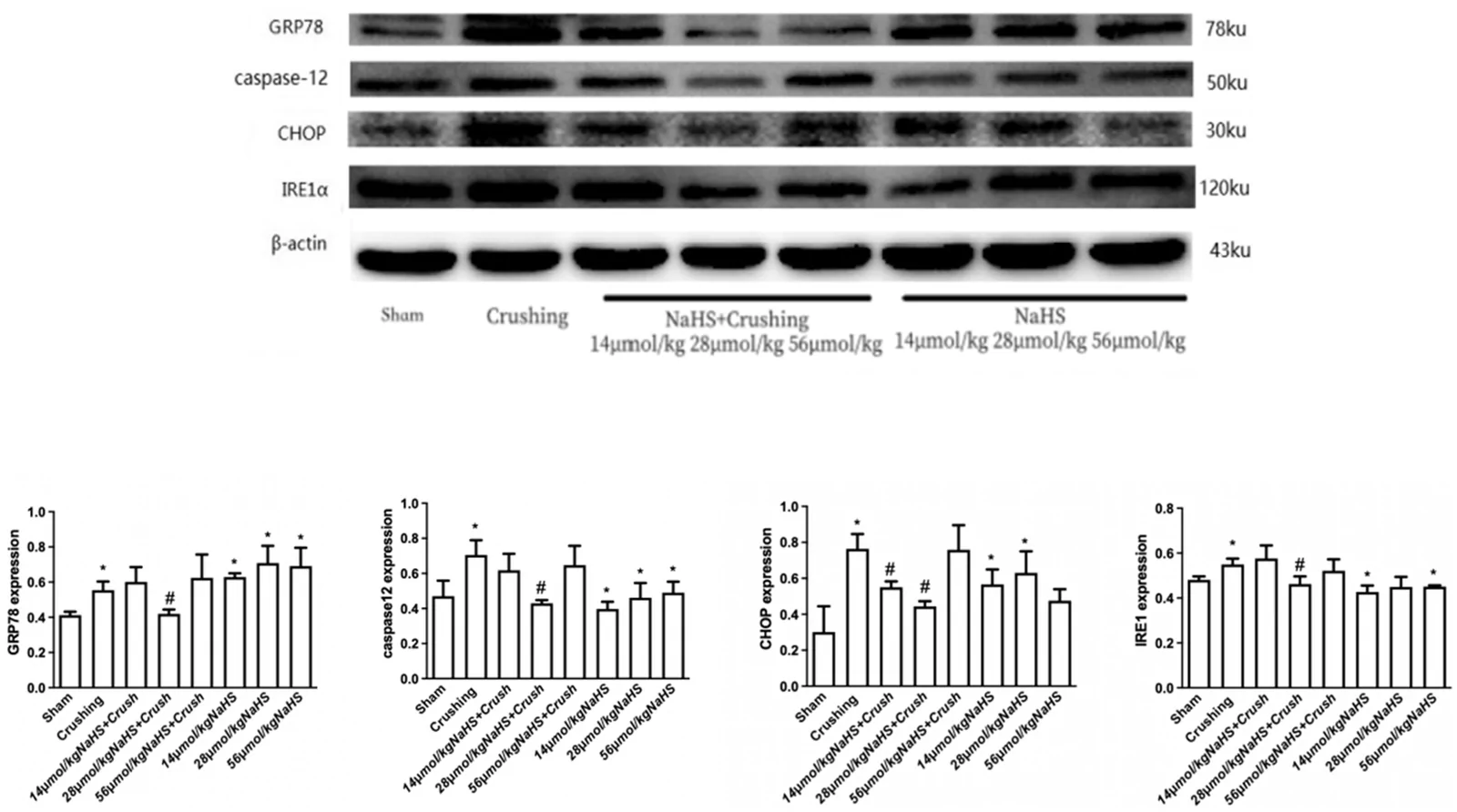
Effects of H_2_S pretreatment on trauma-induced ERS in rat lung tissues. *p<0.05 vs. Sham group; #p<0.05 vs. crushing group.

Effects of post-administration of H_2_S on trauma-induced ERS in rat lung tissues:as shown in Figure 12, compared with the Sham group, the aforementioned ERS-related proteins in the lung tissues of rats in the crushing group were significantly upregulated (p<0.05), with increases of 23 , 42, 86, and 18 % respectively; the caspase-12 and CHOP proteins in the lung tissues of rats in the 120 μmol/kg NaHS group were significantly upregulated, with increases of 52 and 37 % respectively; compared with the crushing group, the expression of the aforementioned ERS-related proteins in the lung tissues of rats in the crushing + 120 μmol/kg NaHS group was significantly downregulated (p<0.05), with decreases of 9 , 46, 29, and 17 % respectively.

**Figure 12:**
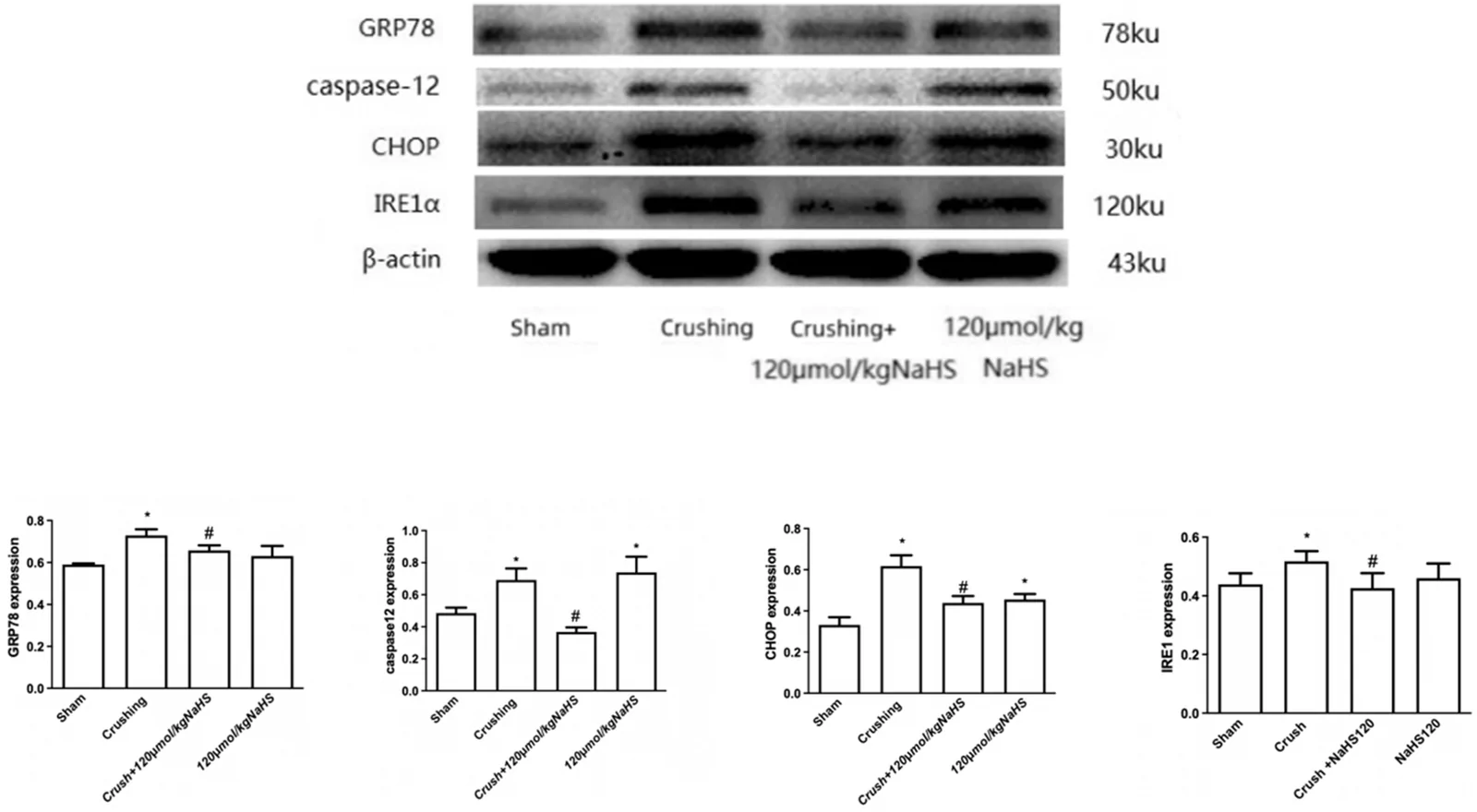
Effects of post-administration of H_2_S on trauma-induced ERS in rat lung tissues. *p<0.05 vs. Sham group; #p<0.05 vs. crushing group.

#### H_2_S can inhibit the increase in HMGB1 protein expression in rat lung tissues induced by traumatic stress

Effects of H_2_S pretreatment on trauma-induced changes in HMGB1 expression in rat lung tissues:as shown in Figure 13, there was a certain amount of basal expression of HMGB1 protein in the lung tissues of rats in the Sham group; compared with the Sham group, the HMGB1 protein in the lung tissues of rats in the crushing group was significantly upregulated, with an increase of 43 %; there was no significant change in the HMGB1 protein in the lung tissues of rats in the 14, 28, and 56 μmol/kg NaHS groups; compared with the crushing group, there was no significant change in the expression of HMGB1 protein in the lung tissues of rats in the 14 and 56 μmol/kg NaHS + crushing groups, while the expression of HMGB1 protein in the lung tissues of rats in the 28 μmol/kg NaHS + crushing group was significantly downregulated, with a decrease of 30 %.

**Figure 13:**
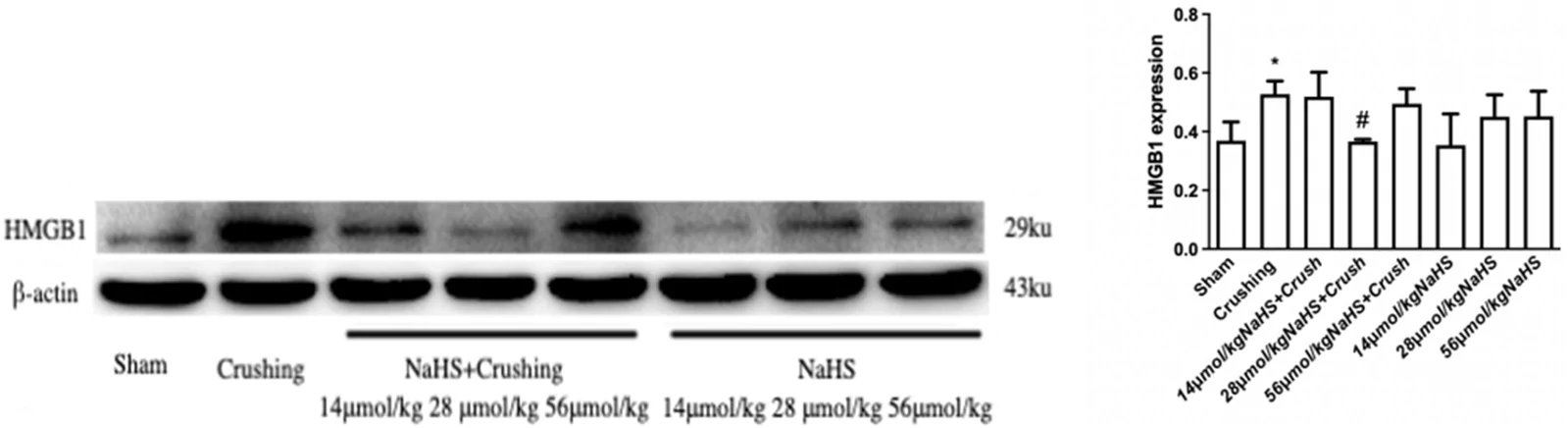
Effects of H_2_S pretreatment on trauma-induced changes in HMGB1 expression in rat lung tissues. *p<0.05 vs. Sham group; #p<0.05 vs. crushing group.

Effects of post-administration of H_2_S on trauma-induced changes in HMGB1 expression in rat lung tissues:as shown in Figure 14,compared with the Sham group, the HMGB1 protein in the lung tissues of rats in the crushing group was significantly upregulated, with an increase of 37 %; the HMGB1 protein in the lung tissues of rats in the 120 μmol/kg NaHS group was significantly upregulated, with an increase of 40 %; compared with the crushing group, the expression of HMGB1 protein in the lung tissues of rats in the crushing + 120 μmol/kg NaHS group was significantly downregulated, with a decrease of 16 %.

**Figure 14:**
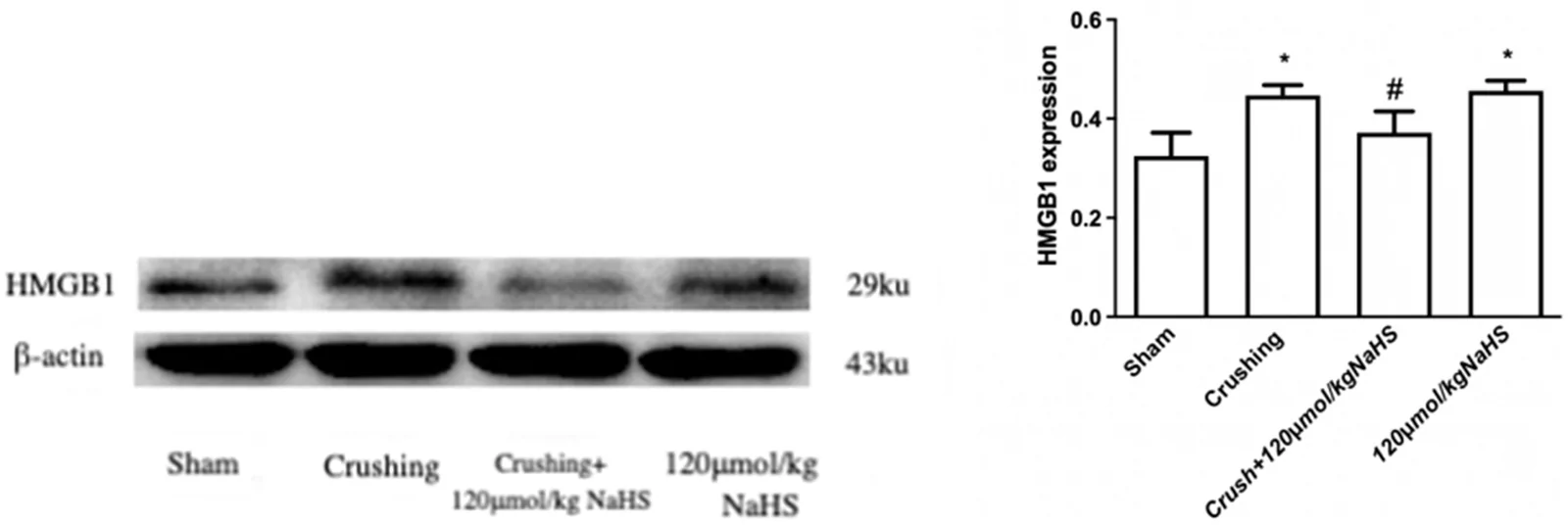
Effects of post-administration of H_2_S on trauma-induced changes in HMGB1 expression in rat lung tissues. *p<0.05 vs. Sham group; #p<0.05 vs. crushing group.

## Discussion

In the present study, we aimed to answer three research questions posed in the Introduction.

First, we asked whether exogenous H_2_S reduces HMGB1 expression/translocation and ERS activation in the liver and lungs of rats subjected to traumatic stress. Our results demonstrate that NaHS pretreatment (28 μmol/kg) significantly lowered plasma HMGB1, reduced HMGB1 cytoplasmic translocation, and downregulated ERS markers (GRP78, caspase-12, CHOP, IRE1α) in both organs.

Second, regarding the dose-response profile of NaHS, we found that 28 μmol/kg pretreatment provided the strongest protection, 14 μmol/kg gave limited protection, 56 μmol/kg showed reduced efficacy with mild toxicity, and delayed administration of 120 μmol/kg also attenuated organ injury but less effectively than 28 μmol/kg pretreatment.

Third, concerning the HMGB1–ERS axis, the protective effect of H_2_S was associated with concurrent suppression of HMGB1 and ERS, suggesting that the HMGB1–ERS pathway may be involved. However, causality remains to be established by future loss-of-function experiments.

### H_2_S alleviates acute hepatic/lung injury caused by traumatic stress

The present study demonstrates that exogenous H_2_S exerts a clear protective effect against traumatic stress-induced acute hepatic and lung injury, with a distinct dose-dependent pattern. Low to moderate NaHS doses (14–28 μmol/kg) effectively reduced biochemical and histopathological indicators of organ damage, whereas the protective effect declined at 56 μmol/kg and cytotoxicity became evident. These findings are consistent with prior reports indicating that H_2_S displays a biphasic, hormetic dose–response profile in multiple pathological models [16], 17]. The optimal efficacy of the 28 μmol/kg dose across several studies suggests that this range may best mimic physiological H_2_S signaling while avoiding toxic accumulation.

Mechanistically, the protective effect is likely related to restoring endogenous H_2_S homeostasis disrupted by traumatic stress. The mechanisms by which H_2_S alleviates hepatic/lung injury caused by various reasons may include: regulating the cellular redox reaction, opening ATP-sensitive potassium channel channels, antagonizing inflammatory reactions [18], and inhibiting the cell apoptosis pathway. The mechanism of hepatic injury caused by severe pathogenic factors such as trauma, hemorrhagic shock [11], and ischemia-reperfusion [10] is related to inhibiting H_2_S production and reducing H_2_S-mediated inflammatory reactions. After supplementing exogenous H_2_S, hepatic injury is alleviated. Previous research results of Cen Xinhai in this laboratory found that compressing the bilateral hindlimbs of rats could lead to significant abnormalities in liver function, accompanied by a decrease in plasma H_2_S content and a decrease in hepatic tissue H_2_S-generating enzyme cystathionine γ-lyase activity. After intraperitoneal injection of H_2_S donor NaHS in advance, it was found that hepatic injury in rats was alleviated [9]^.^ At the level of cultured rat hepatocytes, during lipopolysaccharide (LPS)-induced injury of rat hepatocytes, the content of endogenous H_2_S changes but exerting dose-dependent protective effects, which can alleviate or aggravate cell injury. Administration of an appropriate concentration of exogenous H_2_S can alleviate LPS-induced injury of rat hepatocytes. The mechanism by which exogenous H_2_S alleviates lung injury caused by traumatic hemorrhagic shock is to reduce the effect of oxidative stress. The mechanism by which exogenous H_2_S alleviates LPS-induced acute lung injury in rats is that H_2_S inhibits the expression of pro-inflammatory factors and promotes the expression of anti-inflammatory factors; reduces the expression of apoptotic proteins and enhances antioxidant capacity [19], 20]. For example, a 2021 study reported that the novel H_2_S donor GYY4137 effectively alleviates sepsis-induced acute lung injury by inhibiting the PDGFRβ/Akt/NF-κB/NLRP3 signaling pathway and reducing the expression of related inflammatory factors [21].

### H_2_S inhibits ERS in hepatic/lung tissues of rats caused by traumatic stress

Excessive or prolonged ERS can trigger apoptosis by activating the intrinsic cell death program and has been recognized as a key pathogenic mechanism in various diseases and injury processes [22]. ERS participates in almost all forms of hepatic injury – including immune-mediated, metabolic, viral, and toxic liver injury – and accumulating evidence indicates that it also plays a central role in trauma-related hepatic damage [23]. Similarly, ERS-induced apoptosis contributes to lung injury following myocardial ischemia–reperfusion [24], while the release of neutrophil granules triggered by ERS promotes acute lung injury after intestinal ischemia [25]. Pharmacological inhibition of ERS with 4-phenylbutyric acid (4-PBA) can markedly attenuate trauma-induced hepatic and pulmonary injury [26], further confirming ERS as a critical pathogenic link in remote organ injury following traumatic stress.

Based on our previous findings that H_2_S enhances systemic antioxidant capacity and reduces oxidative damage in distant organs under traumatic stress, we inferred that the upstream mechanism of H_2_S-mediated organ protection likely involves the mitigation of hepatic and pulmonary ERS. However, to date, no studies have directly examined whether H_2_S attenuates traumatic stress-induced acute liver and lung injury via modulation of ERS.

In the present study, pretreatment with exogenous H_2_S donor NaHS effectively reduced ERS in hepatic tissues of rats subjected to compression. Low-dose pretreatment with NaHS alleviated hepatic ERS to varying degrees, with 28 μmol/kg showing the most pronounced effect, followed by 14 μmol/kg, whereas 56 μmol/kg showed only mild improvement. Notably, single administration of low-dose NaHS alone did not induce hepatic ERS, and high-dose NaHS alone only elevated IRE1α, suggesting limited ERS induction. It should be noted that these observations are based on the expression levels of ERS marker proteins (GRP78, CHOP, IRE1α, caspase-12) rather than on functional ERS assays (e.g., XBP1 splicing or ER morphology).Despite the mild inherent toxicity of high-dose NaHS, post-treatment with this dose after compression markedly alleviated hepatic ERS.

In lung tissues, single administration of low-dose NaHS produced a roughly dose-dependent increase in ERS. Pretreatment with 28 μmol/kg NaHS significantly attenuated ERS after compression, whereas the 14 μmol/kg dose reduced only CHOP expression and the 56 μmol/kg dose showed no significant effect. High-dose NaHS alone increased apoptosis-related proteins, but post-treatment after compression significantly reduced lung ERS.As in the liver, these conclusions are drawn from ERS marker protein expression rather than from functional ERS readouts.

Collectively, these results indicate that low-dose NaHS alone induces little to no ERS in liver and lung tissues, yet pretreatment followed by compression leads to marked ERS attenuation – most effectively at 28 μmol/kg. Although high-dose NaHS exhibits some inherent cytotoxicity and can induce ERS on its own, high-dose post-treatment after compression still produced substantial reductions in hepatic and pulmonary ERS.

Our findings confirm that appropriately dosed H_2_S can attenuate ERS in distant organs under traumatic stress. Nevertheless, the precise mechanisms by which H_2_S reduces ERS remain unclear. Previous work from our laboratory demonstrated that HMGB1 functions as an upstream regulator of ERS and mediates traumatic stress-induced acute liver and lung injury [7], 8]. This raises an important mechanistic question: Does H_2_S attenuate traumatic stress-induced ERS by suppressing HMGB1?

### H_2_S inhibits the increased expression of HMGB1 in hepatic/lung tissues and the increase in HMGB1 content in blood caused by traumatic stress

HMGB1 is a nuclear DNA-binding protein that can be actively secreted or passively released under stress, functioning as a potent damage-associated molecular pattern [27]. Its expression and extranuclear translocation increase in various forms of liver and lung injury, acting not only as a “late” inflammatory mediator but also as an “early” driver of sterile inflammation [28], 29]. Accumulating evidence indicates that HMGB1 contributes to distant-organ injury following traumatic stress, and that both ER stress (ERS) and HMGB1 serve as key pathological nodes [23]. However, the interplay between HMGB1 and ERS in trauma-induced hepatic/lung injury has not been clearly defined.

Our concurrent work demonstrated that suppression of HMGB1 up-regulation alleviates trauma-induced ERS, supporting HMGB1 as an upstream activator of the ERS pathway [23]. Previous studies in this laboratory showed that reduced endogenous H_2_S participates in trauma-induced hepatic injury, and exogenous H_2_S confers protection [3]. Whether this protection involves modulation of HMGB1 has not been previously reported.

In the present study, ELISA results showed that although single injections of NaHS at different concentrations increased plasma HMGB1 to varying degrees, exogenous H_2_S markedly inhibited trauma-induced HMGB1 elevation, with 28 μmol/kg pretreatment producing the strongest effect. Western blotting showed that HMGB1 expression in hepatic/lung tissues was significantly reduced in the 28 μmol/kg pretreatment and 120 μmol/kg post-treatment groups, but not in the 14 or 56 μmol/kg pretreatment groups. Single injections of 14 or 28 μmol/kg NaHS alone caused no change in tissue HMGB1, whereas 56 μmol/kg elevated hepatic HMGB1 and 120 μmol/kg elevated both hepatic and pulmonary HMGB1. IHC confirmed that the 28 μmol/kg pretreatment and 120 μmol/kg post-treatment groups most effectively prevented HMGB1 translocation induced by compression.

Overall, low-dose NaHS (14–28 μmol/kg) exhibited minimal cytotoxicity and little effect on baseline HMGB1, whereas 56 μmol/kg caused mild cytotoxicity with HMGB1 elevation, and 120 μmol/kg exerted stronger cytotoxicity with further increases. The reduced protective effect of 56 μmol/kg likely reflects the biphasic nature of H_2_S: low to moderate doses are cytoprotective, whereas higher concentrations can induce mild cellular stress, disrupt mitochondrial function, increase oxidative stress, and partially offset protection. In line with this, Feng et al. demonstrated that 3 and 5 mg/kg NaHS (∼56–93 μmol/kg for a 200 g rat) inhibited mitochondrial complex IV activity, increased ROS generation, and induced liver apoptosis, while 1 mg/kg (∼19 μmol/kg) had no detectable toxic effect on the liver [30]. In contrast, 120 μmol/kg post-treatment remains effective because, after trauma, tissues experience oxidative stress and inflammation, necessitating a higher H_2_S dose to suppress HMGB1 up-regulation and alleviate ER stress.

Plasma HMGB1 elevation mainly resulted from necrotic muscle cells releasing HMGB1 into circulation. Circulating HMGB1, together with other trauma-induced mediators, may activate non-parenchymal hepatic and pulmonary cells to further secrete HMGB1, creating a feed-forward loop that exacerbates distant-organ injury.

### Mechanistic speculation on H_2_S regulating HMGB1/ERS pathway

Literature review shows that the reasons for the active secretion of HMGB1 include post-translational modification, ROS signaling pathway, Ca^2+^ signaling pathway, NO signaling pathway, NF-κB pathway, MAPK pathway, PARP-dependent mechanism, etc.; passive release is due to cell death caused by various external factors, including necrosis, apoptosis, cell lysis, and autophagy. Among the above reasons, H_2_S may affect the translocation of HMGB1 by influencing these causes of release or secretion, thereby affecting its function. It is particularly worth noting that H_2_S, as an important redox regulator, may cause sulfhydration modification of sensitive sulfhydryl groups (-SH) at positions 23, 45, and 106 of cysteine in HMGB1, changing the biological characteristics of HMGB1 and its pro-inflammatory effect. Since HMGB1 requires the help of multiple receptors to achieve signal transduction, among which TLR-like and RAGE receptors are the most widely studied [31], H_2_S may change its binding to receptors after sulfhydration modification of HMGB1, thereby affecting the downstream signal transduction of HMGB1. In addition, H_2_S may also prevent HMGB1 from binding to receptors by affecting the HMGB1 receptors themselves. Recent studies have found that the secretion and function of HMGB1 are finely regulated by post-translational modifications such as acetylation, phosphorylation, and thiol oxidation. These modifications determine whether HMGB1 translocates from the nucleus to the cytoplasm and is subsequently secreted extracellularly, which is a key mechanism underlying its role in inflammatory responses [32], [33], [34].Although the above mechanisms offer plausible explanations for the observed effects, the current study did not directly assess whether H_2_S-induced sulfhydration of HMGB1 occurs in this traumatic stress model. Therefore, the proposed mechanisms remain speculative and require further investigation, including biochemical assays such as biotin switch assays or mass spectrometry, to validate the involvement of HMGB1 sulfhydration in the protective effects of H_2_S.

### Conclusion and prospect

In conclusion, exogenous H_2_S significantly alleviates traumatic stress-induced acute liver and lung injury in rats. This protection is associated with reduced HMGB1 expression/cytoplasmic translocation and suppressed endoplasmic reticulum stress (ERS) activation. Given that our concurrent studies demonstrate that the HMGB1-ERS pathway mediates trauma-induced organ injury, the present findings suggest that exogenous H_2_S may exert its protective effects, at least in part, through inhibition of the HMGB1-ERS axis. Furthermore, our previous work showing that decreased endogenous H_2_S production contributes to acute liver injury after trauma raises the possibility that downregulation of the HMGB1-ERS pathway may underlie the loss of endogenous protection.

Several limitations should be acknowledged. First, this study used only male rats in an acute (18 h) hindlimb crush model, which limits generalizability to other species, sexes, or chronic injury settings. Second, we lack direct measurements of HMGB1 post-translational modifications (e.g., acetylation, phosphorylation) or S-sulfhydration. Third, receptor-level readouts (e.g., TLR4, RAGE) are not available in the current data. Fourth, our current data do not distinguish whether HMGB1 acts upstream of ERS or whether both pathways are regulated in parallel during traumatic stress.

Future work will include pharmacological inhibition (glycyrrhizin for HMGB1, 4-PBA for ERS) and gene silencing experiments to determine whether disruption of the HMGB1-ERS axis abolishes the protective effect of H_2_S. Additionally, as an important redox regulator, H_2_S may influence post-translational modifications of HMGB1 (acetylation, ADP-ribosylation, methylation, phosphorylation, glycosylation, oxidation) and induce S-sulfhydration of its cysteine residues (Cys23, Cys45, Cys106), thereby altering HMGB1’s biochemical properties and pro-inflammatory activity. These mechanistic possibilities warrant further investigation using biochemical assays such as biotin switch or mass spectrometry.
